# Overcoming target epitope masking resistance that can occur on low-antigen-expresser AML blasts after IL-1RAP chimeric antigen receptor T cell therapy using the inducible caspase 9 suicide gene safety switch

**DOI:** 10.1038/s41417-020-00284-3

**Published:** 2021-01-07

**Authors:** Walid Warda, Mathieu Neto Da Rocha, Rim Trad, Rafik Haderbache, Yahya Salma, Lucie Bouquet, Xavier Roussel, Clémentine Nicod, Marina Deschamps, Christophe Ferrand

**Affiliations:** 1 INSERM UMR1098 Right, EFS BFC, Univ. Bourgogne Franche-Comté, 25000 Besançon, France; 2 Laboratory of Applied Biotechnology (LBA3B), AZM Center for Research in Biotechnology and its Applications, Lebanese University, Tripoli, 1300 Lebanon; 3 Hematology Department, Hôpital Jean Minjoz, 25000 Besançon, France

**Keywords:** Cancer immunotherapy, Leukaemia, Gene therapy

## Abstract

Although chimeric antigen receptor CAR) T cell immunotherapies are an undeniable and unequivocal success, knowledge obtained from the monitoring of the first clinical trials targeting the CD19 antigen in B malignancies, either refractory/relapsed acute lymphoid leukemia (ALL) or lymphomas, contributed to the identification of tumor cell escape in about 30–50% of B-ALL. Resistance occurred due to loss of surface expression of the antigen (rCD19−) or to the early disappearance or inactivation of CAR T cells (rCD19+). In a recently reported clinical case, rCD19− relapse resulted from masking of the antigen by the CAR at the surface of B-ALL leukemia cells following the unexpected viral transduction of a leukemic cell present in the cytapheresis sample. The objective of this work was to reproduce this epitope-masking resistance model, in the context of acute myeloid leukemia (AML), based on our immunotherapeutic CAR T cell model targeting the accessory protein of the interleukin-1 receptor (IL-1RAP) expressed by leukemic stem cells. As AML primary blasts express different levels of IL-1RAP, we modeled transduction of different AML tumor cell lines screened for density of antigenic sites with our lentiviral vectors carrying a third-generation IL-1RAP CAR, an iCASP9 suicide gene, and a truncated CD19 surface gene. We demonstrated that primary AML blasts can be easily transduced (74.55 ± 21.29%, *n* = 4) and that CAR T cytotoxicity to IL-1RAP is inversely correlated with epitope masking in relation to the number of antigenic sites expressed on the surface of IL-1RAP+ lines. Importantly, we showed that, in vitro, a 24-h exposure of IL-1RAP+/CAR+ leukemia lines to Rimiducid eliminated >85% of the cells. We confirmed that the expression of IL-1RAP CAR by an IL-1RAP+ leukemic cell, by decreasing the membrane availability of the targeted antigen, can induce resistance while a high epitope density maintains sensitivity to CAR T cells. Moreover, the presence of the iCASP9/Rimiducid suicide system safety switch makes this immunotherapy approach safe for application in a future phase 1 clinical trial.

## Background

Innovative adoptive chimeric antigen receptor (CAR) T cell therapy has demonstrated accepted and impressive results in the treatment of hematologic B malignancies, particularly in refractory/relapse (R/R) pediatric or adult acute lymphoid leukemia (ALL) [1] and lymphoma [2] by targeting the CD19 cell surface antigen. Based on these pivotal clinical trials, in which the short-term complete remission (CR) rate has reached 70–90%, two autologous CAR T cell drugs have been approved by the Food and Drug Administration. However, careful retrospective monitoring follow-up has shown that 20–50% of CR patients relapse within the first year and that 10–20% of patients do not achieve CR [3].

Intention-to-treat analysis has revealed several major factors impairing CAR T cell immunotherapy [4]. The first is CAR T cell manufacturing failure, starting from a low-quality apheresis harvest of T cells, either in too small quantities or of inappropriate intrinsic quality from patients who have received multiple treatments depending on the disease’s history, and with production delay that mismatches the progression of the patient’s acute disease [5].

The second problem is primary resistance, independent of target antigen expression (antigen-positive relapse), which affects CAR T cell efficiency early after infusion due to CAR T cell persistence (costimulatory domains), T cell subpopulation composition (naive versus memory T cells), immune checkpoint expression, and negative interaction with regulatory T cells or myeloid-derived suppressor cells [6, 7].

The third reason is relapse, mainly due to loss of membrane antigen target expression (antigen-negative relapse, Ag−/R). Several mechanisms have been identified as immune pressure inducing a lineage switch from ALL to a myeloid phenotype that occurs in MLL-rearranged B-ALL [8], including homozygous mutations of the B cell receptor protein complex [9]; splicing or mutations, respectively, of the CD19 mRNA or gene [10–12]; and tumor/CAR T cell membrane exchange that decreases antigen density and induces fratricidal CAR T cell killing (trogocytosis) [13]. Another reported but unusual Ag−/R CAR T cell escape mechanism is epitope masking following unintentional viral transduction of a single leukemic B cell during CAR T cell production, leading to autorecognition of the target by tumor-expressed CAR, which makes it invisible to the CAR T cells [14].

There are various ways to overcome antigen escape and avoid failure in CAR T cell immunotherapy [15], such as improving CAR T cell production (using interleukin (IL)-7 and IL-15 rather than IL-2), combining the therapy with anti-checkpoint inhibitor antibodies, targeting an alternative cell surface epitope (i.e., CD20, CD22, etc.) expressed by the same tumor cells using dual- or tandem-specificity CARs [16], using a universal CAR [17], or armoring the CAR construct to enhance its antitumor activity [18].

Regarding epitope masking, anti-CAR-CAR T cell therapy was recently proposed as an antidote in order to eliminate transduced leukemic B cells [19]. Although useful for treating this unintended consequence of CD19 CAR T cell strategies, it is not applicable to CAR T cells directed against other targets, and much effort and time are required to implement such an autologous approach. Thus there is a need to explore other strategies. Suicide gene safety switches [20, 21] may be a universal and interesting solution to explore while it can also secure at the same time either adverse events due to CAR T cells or but also unexpected and accidental tumor cell transduction.

We present a proof of concept for a CAR T cell immunotherapy approach to targeting chronic myeloid leukemia (CML) or acute myeloid leukemia (AML) leukemic stem cells that express interleukin-1 receptor accessory protein (IL-1RAP) [22]. The method takes advantage of the inducible caspase 9 (iCASP9) suicide gene [23] in our lentiviral construct. Since we plan to use this immunotherapy in the clinic, we modeled the epitope masking and studied the usefulness of the suicide safety switch in the context of AML.

## Methods

### Patient samples, healthy donor blood samples, and cell lines

Samples were collected from patients with AML who are included in the French Innovative Leukemia Organization Cell Biobank, AML collection (No. BB-0033-00073, declaration 2009-944 and authorization AC 201261739). Peripheral blood mononuclear cells were isolated by Ficoll gradient density centrifugation using Ficoll–Paque from anonymous blood samples collected from healthy donors at the French Blood Center (Besançon, France). Patients and donors provided written informed consent, and the study was conducted in accordance with the ethical guidelines (Declaration of Helsinki) and approved by the local ethics committee of the CPP-Est (CPP2019-03-022, France).

Human tumor cell lines KU812 (CRL-2099), K562 (CCL-243), HEL (ACC-11), and MA9-RAS were kindly provided by James C. Mulloy, Division of Experimental Hematology and Cancer Biology, Cincinnati Children’s Hospital Medical Center, University of Cincinnati College of Medicine, MOLM-13 (ACC-554), MONO-MAC-6 (ACC-124), EOL-1 (ACC-386), HL-60 (UMR645), THP-1 (ACC-16), and 293T (CRL-3216) were obtained from the ATCC or DMSZ and stored in our local master cell bank. A derived working cell bank was use for all experiments in this work.

### IL-1RAP CAR T and AML primary cell identification by flow cytometry

Leukemic cells from patients with AML were tracked using a panel containing the following antibodies: CD45-V50 (HI30, BD Biosciences), CD34-V450 (8G2, BD Biosciences), CD38-APC (HB-7, BD Biosciences), CD33-PerCP-Cy5.5 (P67-6, BD Biosciences), CD14-APC-H7 (MφP9, BD Biosciences), and our murine fluorescein isothiocyanate (FITC)-labeled IL-1RAP monoclonal antibody (mAb), clone #A3C3. Transduced cells were stained using antibodies CD3-vioblue (clone REA613, Miltenyi Biotec) and CD19-APC (LT19, Miltenyi Biotec). Cells were collected using a CANTO II cytometer (BD Biosciences) and analyzed using the DIVA 6.1 software (BD Biosciences).

### Surface IL-1RAP antigen quantification by flow cytometry

K562, KU812, HEL, HL-60, THP-1, MA9RAS, Molm-13, EOL-1, and Mono-Mac-6 cells were stained for indirect immunofluorescence with specific mAbs and anti-IL-1RAP and analyzed by quantitative flow cytometry. The saturating concentration of primary antibody was previously determined for each cell line according to the manufacturer’s instructions.

IL-1RAP expression levels were determined using indirect immunofluorescence flow cytometry using the Cell Quant Calibrator (Biocide, France). Briefly, cells were labeled with unconjugated anti-IL-1RAP mAb and the binding of the primary antibody was revealed using a FITC‐coupled secondary antibody, anti-IgG1 (clone X-56, Miltenyi Biotec). Mean of fluorescence intensity (MFI) calibration values for the calibration beads and the corresponding numbers of mAb molecules were determined. The MFI value of the test sample was compared with the calibration curve to determine the number of bound mAb molecules.

### Lentiviral constructs and cell transduction by IL-1RAP CAR vector

The IL-1RAP CAR lentiviral construct was generated as previously described [22]. Briefly, this vector carries a third-generation CAR, an iCASP9 (inducible caspase 9) suicide gene, and a gene encoding surface-expressed delta CD19 for monitoring transduction efficiency and CAR T cell follow-up. The mock control vector does not contain the CAR sequence.

Transduced cells were established from T cell lines, primary T cells from healthy donors, or primary cells from AML patients and transduced with lentiviral supernatant from IL-1RAP CAR- or mock-transduced cells. IL-1RAP CAR T cells, mock-transduced T cells, and untransduced T cells were then used in functionality tests, in order to subtract alloreactivity effect that may occur.

Briefly, T cells were activated using CD3/CD28 magnetic beads (Life Technologies) at a 1∶1 ratio in X-vivo15 medium (Lonza) with 500 UI/mL of IL-2 (Proleukin, Novartis) for 2 days, transduced with the CAR-expressing lentivirus, and finally expanded for up to 10 days. CD3+ cells were selected using CD3/CD28 magnetic beads. AML primary cells or cell lines were transduced prior to CD3/CD28 activation or selection.

For all transduced cells, the transduction efficiency was established by use of CD19 staining with an allophycocyanin (APC)-conjugated anti-CD19 antibody (clone LT19, Miltenyi Biotec) and flow cytometry. CAR cell-surface expression was determined using a biotinylated IL-1RAP protein, phycoerythrin-conjugated streptavidin (Miltenyi Biotec), and flow cytometry (BD CantoII).

### In vitro CAR T cell cytotoxicity analysis

Untransduced-, mock-transduced, or IL-1RAP CAR T cells were labeled with the cell proliferation dye eFluor 450 (ThermoFisher, Switzerland) following the manufacturer’s protocol. Labeled cells were cultured at an appropriate effector-to-target (E:T) ratio with untransduced, mock+, or CAR+ target AML cell lines at 37 °C for 24 h. After co-culture, cells were labeled with 7-aminoactinomycin D (7-AAD; Beckman Coulter, USA) and anti-CD19-APC to evaluate the number of dead (7-AAD positive) target cells.

### IL-1RAP tumor xenografted murine models

A xenograft NOD.Cg-Prkdc^scid^ Il2rg^tm1Wjl^ Tg (CMV-IL3,CSF2,KITLG) NSG-SGM3 (NSGS) murine model was used to study CAR T cell cytotoxicity and iCASP9/Rimiducid suicide gene efficiency. NSGS mice were transplanted (intravenously (i.v.)) with bulk Luc+ IL-1RAP+ AML cells that were unmodified or modified with IL-1RAP CAR vector.

HL-60 and IL-1RAP CAR+ HL-60 AML cell lines were transduced with a luciferase lentiviral vector (pLenti CMV V5-Luc Blast vector, Addgene), and luciferase-positive cells were selected by resistance to blasticidin (ThermoFisher).

Six-to-8-week-old NSG-S mice (Jackson Laboratory) were sublethally irradiated (250 cGy) on day −4. On day −3, each mouse received 10^6^ HL-60 Luc+ or HL-60 Luc+/CAR+ cells resuspended in 300 µL of phosphate-buffered saline (PBS) by injection into the tail vein. After tumor establishment (day 0), mice were treated with untransduced or IL-RAP CAR T cells (10^7^ cells resuspended in 300 µL of PBS) via the tail vein. An untreated group was used as a control. Tumor development was monitored every week, using an IVIS Lumina III system (PerkinElmer) after injection of 3 mg of luciferin (VivoGlo Luciferin, Promega, USA) intraperitoneally (i.p.) within 10 min of imaging.

Mice were monitored until the animals in the untreated group reached a moribund state and manifested signs of leukemia (i.e., weight loss >15%, decreased activity, and/or hind limb paralysis). Animal protocols were performed under the control of the Animal Care and Use Committee of the University of Besançon (CELEAG and protocol 11007R, Veterinary Services for Animal Health & Protection).

### In vitro and in vivo iCASP9 safety switch functionality

In order to test the functionality of the iCSP9 safety switch, IL-1RAP CAR-positive AML cell lines or AML patient-derived xenografted (PDX) blasts were cultured in medium alone or containing 20 nM of dimerizer (AP1903, Rimiducid, Bellicum Pharmaceutical, Houston, USA). Untransduced cell lines were used for controls. After 24 h, cells were transferred to Trucount tubes (BD Biosciences) and stained with anti-CD19-APC, anti-annexin-V-FITC (Miltenyi), and 7-AAD for flow cytometry. Fluorescence analysis was gated on CD19-positive cells. Cells were considered viable when they were negative for both annexin-V and 7-AAD. The quantification was performed as previously described [23].

To evaluate the functionality of the suicide gene in vivo, mice engrafted with HL-60 or HL-60 CAR+ cells were treated with Rimiducid (50 µg, i.p.) [24]. Control mice received a dose of PBS. Mice were then imaged as described above.

## Results

### Primary AML leukemic cells expressing different levels of IL-1RAP can be easily transduced using IL-1RAP lentiviral vector supernatant

**Fig. 1:**
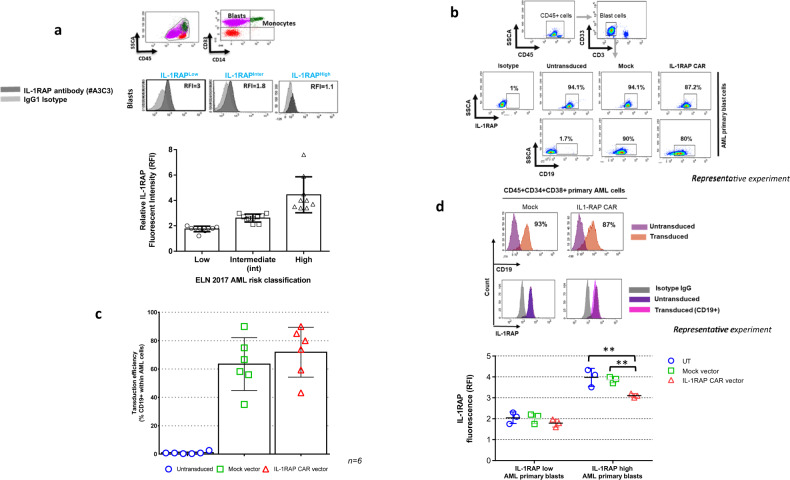
Transduction of primary AML cells and IL-1RAP cell surface expression. **a** Upper: representative flow cytometric analysis of AML primary cells from blood. The IL-1RAP histogram is shaded dark gray. Relative fluorescence intensity (RFI) is provided and calculated relative to the intensity of IgG1 isotype staining (light gray). Blast cells were characterized and discriminated from monocytes by their CD45+CD34+CD38+CD14− phenotype in flow cytometry. Lower: distribution of primary blasts from AML patients (*n* = 30) in 3 groups according to the intensity of their cell surface IL-1RAP staining (RFI). 1 ≤ RFI low ≤ 2 (*n* = 9); 2 < RFI inter ≤ 3 (*n* = 13); 3 < RFI high (*n* = 8). **b** Representative cytometry gating analysis of the transduction efficiency of primary AML blast cells with the mock or CAR lentiviral vector carrying the ∆CD19 gene. After 48 h of transduction, untransduced or transduced cells were stained with anti-CD19-APC and the percentage of CD19+ cells was measured by flow cytometry. Blasts were gated on CD45+ cells and then discriminated from T cells and monocytes. **c** Results of transduction efficiency of primary AML cells are presented as percentage mean ± SD of seven independent experiments from six independent starting AML samples. **d** Upper: detection of IL-1RAP expression on the surface of primary AML cells. Untransduced or transduced (mock or CAR) cells were stained using anti-CD19-APC and anti-IL-1RAP-FITC and evaluated by flow cytometry. An isotype-matched IgG was used as a negative control. **d** Lower: relative fluorescence intensity of IL-1RAP cell surface expression, AML primary blasts of 6 patients (3 low and 3 high IL-1RAP expresser primary blasts) untransduced (blue circles), or transduced with either mock (green squares) or IL-1RAP CAR (red triangles) vectors. **p < 0.01.

Primary AML blasts express different levels of IL-1RAP [25]. We confirmed that finding on primary AML blasts (*n* = 30) using cytometry with our own monoclonal antibody (#A3C3) used for CAR design. Samples representing European Leukemia Net classification groups equally (*n* = 10 in each group) expressed different levels of IL-1RAP as shown in a representative experiment and as reported using relative fluorescence intensity (RFI). IL-1RAP low expressers are defined by overlapping isotype control histogram (Fig. 1a).

In the absence of T cell selection, leukemic AML primary cells could be transduced by our IL-1RAP CAR and mock vectors with a high efficiency of transduction (71.9 ± 17.6% and 63.5 ± 18.66% for CAR and mock vectors, respectively, *n* = 6; Fig. 1b, c).

Moreover, the intensity of IL-1RAP expression was slightly decreased on the surface of leukemic AML primary cells transduced by the CAR vector and remained unchanged after transduction with the mock vector in comparison with untransduced primary blast cells (Fig. 1d, upper).

IL-RAP cell surface expression decrease was also shown on primary blasts (low and high IL-1RAP expressers, *n* = 6) from AML patients, with a clear difference in high IL-1RAP expressers compared to low IL-1RAP expressers (Fig. 1d, lower).

### Lentiviral IL-1RAP CAR transduction of AML cell lines expressing different levels of IL-1RAP affects IL-1RAP detection

**Fig. 2:**
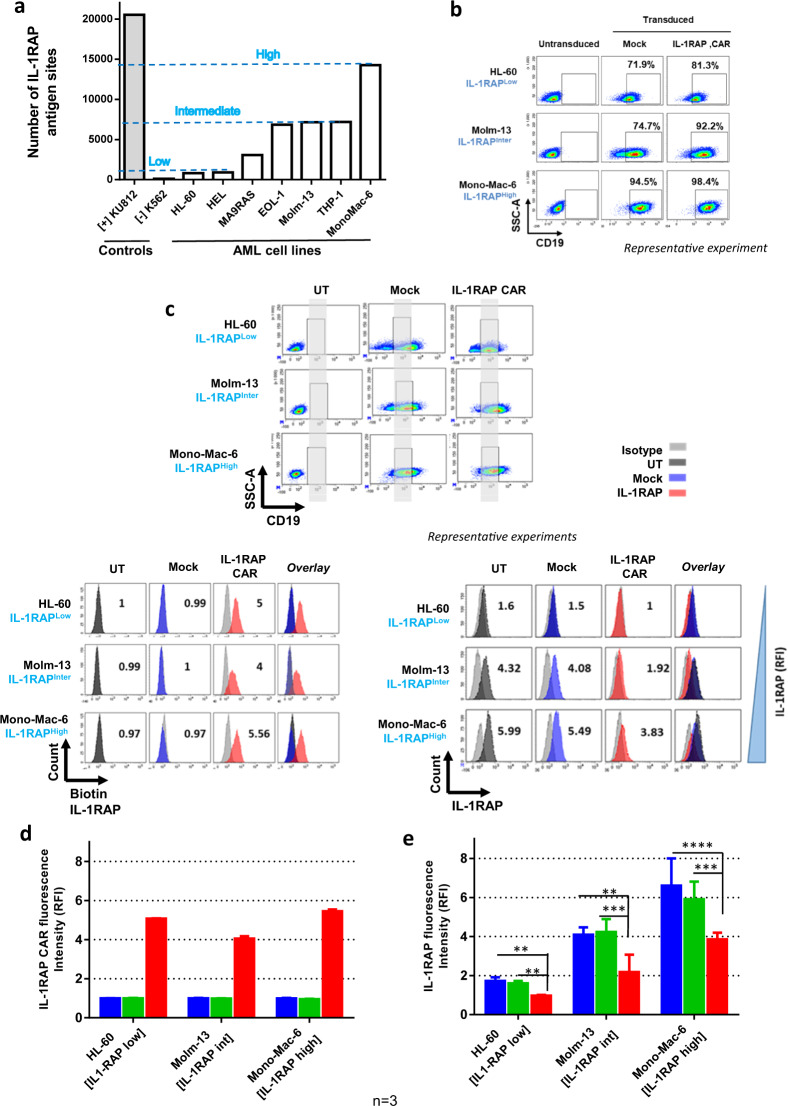
Generation of CAR-transduced leukemic cell lines. **a** Determination of the absolute number of IL-1RAP antigenic sites on the surface of seven different AML cell lines by flow cytometry using calibration beads, using anti-IL-1RAP antibody and a secondary anti-mouse antibody coupled to FITC. Three groups were defined by IL-1RAP expression level: IL-1RAP^low^ (HEL, HL-60, and MA9RAS), IL-1RAP^inter^ (EOL-1, Molm-13, and THP-1), and IL-1RAP^high^ (Mono-Mac-6 and KU812). K562 and KU812 cell lines were used as negative and positive controls, respectively. **b** Production of CAR+ leukemic AML cell lines. Cell lines belonging to the three IL-1RAP expression-level groups were transduced with mock or CAR vector. Transduction efficiency, provided as a percentage, was measured by flow cytometry using anti-CD19-APC, representing cell surface CD19 expression on transduced cells. Untransduced cells were used as a negative control. **c** Upper: normalization by gating on UT, Mock, or CAR transduced cell line subpopulations based on similar CD19 cell surface expression (grey area). Left lower: CAR expression at the cell surface of CAR-transduced leukemic cells. Representative experiment: cells were incubated with biotinylated recombinant IL-1RAP protein and stained using streptavidin-PE by flow cytometry. RFI of IL-1RAP CAR staining relative to UT cells is provided. Dark gray represents the untransduced cells. Light gray, blue, or red histograms correspond, respectively, to matched isotype staining or Biot-IL-1RAP staining of Mock- or CAR-transduced cell lines. Right lower: relative cell surface expression of IL‐1RAP on AML cell lines HL‐60, Molm‐13, and Mono‐Mac‐6 before (untransduced = UT) or after transduction with mock or CAR vectors determined by staining cells with anti‐IL‐1RAP‐FITC and flow cytometry. An isotype-matched IgG‐FITC was used as a negative control. MFI (mean of fluorescence intensity) is provided. **d** Relative expression of IL-1RAP CAR cell surface expression, expressed as relative fluorescence intensity (RFI) on the three different cell lines before (untransduced, blue histograms) or after transduction with mock (green histograms) or CAR vector (red histograms) determined by staining cells with anti-IL-1RAP-FITC and flow cytometry. **e** Relative expression of IL-1RAP cell surface expression, expressed as relative fluorescence intensity (RFI) on the cell lines HL-60 (IL-1RAP low), Molm-13 (IL-1RAP int), and Mono-Mac-6 (IL-1RAP high) cells before (untransduced, blue histograms) or after transduction with mock (green histograms) or CAR vector (red histograms) determined by staining cells with anti-IL-1RAP-FITC and flow cytometry. Analysis was performed by gating on similar CD19 expresser AML subpopulations. An isotype-matched IgG-FITC was used as a negative control. Graph represents four independent experiments. **p < 0.05, ***p < 0.01, ***p < 0.001.

In order to model CAR transduction of various cells expressing surface IL-1RAP, we used IL-1RAP RFI and absolute number of antigen site determination to screen different AML cell lines for IL-1RAP epitope expression. We classified AML cell lines into three types: low [<1000], intermediate [>1000 and <7200], and high [>7200], (IL-1RAP^low^, IL-1RAP^inter^, and IL-1RAP^high^, respectively) according to the quantification of IL-1RAP antigenic sites by flow cytometry (Fig. 2a). For further experiments, we selected HL-60 (IL-1RAP^low^), Molm-13 (IL-1RAP^inter^), and Mono-Mac-6 (IL-1RAP^high^) cell lines as representative models of the different levels of IL-1RAP expression. As shown in Fig. 2b, all three selected cell lines were transduced with the same efficiency.

The autocrine expression of CAR on the surface of cell lines after lentiviral transduction was confirmed by flow cytometry using the biotinylated IL-1RAP protein for all CAR+ AML cell lines (Fig. 2c, right up). In order to exclude a differential effect due to different transduction efficacy, normalization was performed by gating on subcellular population expressing similar CD19 cell surface. Interestingly, by comparison with untransduced cell lines, the intensity of IL-1RAP signal at the cell surface decreased significantly after CAR expression on HL-60 [fold change (FC) = 0.67; *p* < 0.01] on CAR+ Molm-13 (FC = 0.67; *p* < 0.01), and CAR+ Mono-Mac-6 (FC = 0.52; *p* < 0.01) transduced cells, while it remained stable between mock- transduced and their respective untransduced AML cell lines (FC = 0.98; 0.92, and 1.07, respectively, for HL-60, Molm-13, and Mono-Mac-6) (Fig. 2c, right down and Fig. 2d).

### The cytotoxicity of CAR T cells against AML cell lines is reduced only against an IL-1RAP^low^-transduced leukemic AML cell line

**Fig. 3:**
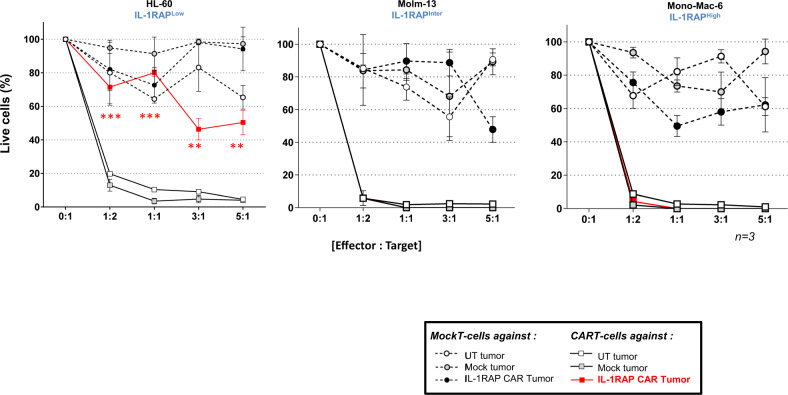
In vitro cytotoxicity of IL-1RAP CAR T cells against CAR+ leukemic cells. Untransduced, mock (circle, dotted line), or CAR T cells (square, solid line) as effector cells were labeled with eFluor and co-cultured with each cell line (HL-60, Molm-13, and Mono-Mac-6 cells) untransduced (UT tumor, white symbols) or transduced with mock (mock tumor, gray symbols) or CAR (CAR tumor, black/red symbols) vectors, at various effector:target ratios. Effector cytotoxicity is reported as the percentage of remaining living cells, gated in cytometry as eFluor−/7-AAD−, normalized to UT effectors. Results are presented as mean ± SD of three independent experiments. Analysis was performed by gating on similar CD19 expresser AML subpopulations. Comparison of IL-1RAP CAR T cells cytotoxicity against UT or Mock versus IL-1RAP CAR+ transduced low IL-1RAP HL-60 AML tumor cell lines: **p < 0.05, ***p < 0.01.

The cytotoxicity of IL-1RAP CAR T cells was tested in vitro against the three AML cell lines HL-60, Molm-13, and Mono-Mac-6 transduced or not with mock or IL-1RAP CAR vectors. Compared with mock-T cells, after 24 h of co-culture at different E:T ratios, IL-1RAP-CAR T cells induced an equivalent mortality in IL-1RAP^high^ and IL-1RAP^inter^ CAR+ AML cell lines (88 and 76%, respectively), while the IL-1RAP^low^ CAR+ AML cell line was significantly less sensitive (57%) even when increasing at an E:T ratio of 5:1 (Fig. 3).

**Fig. 4:**
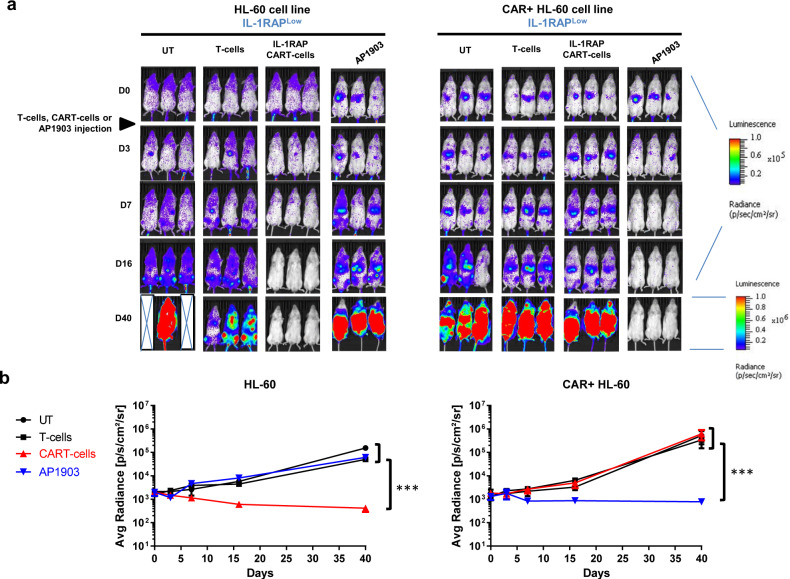
In vivo CAR T cell experimental murine model of xenograft IL-1RAP CAR+ HL-60 AML cell line. Immunodeficient NSG-S mice were irradiated (2.5 Gy) 1 day before injection of 10^6^ luciferase HL-60 or luciferase IL-1RAP CAR+ HL-60 cells. **a** After AML tumor engraftment, mice were left untreated (UT), treated with AP1903 (50 μg i.p.), treated with control T cells (mock), or treated with IL-1RAP CAR T cells (10^7^ cells). From day 2 after tumor infusion, mice were monitored for tumor progression until day 40 by bioluminescence imaging (BLI). (×) dead mice. (▶) the time of injection of untransduced T cells or CAR T cells. Radiance scale is provided for D0 to D16 and for D16 to D40. **b** Radiance curves of the in vivo bioluminescent signal (radiance p/s/cm^2^/sr) recorded using BLI from the time of injection of IL-1RAP CAR T cells at D0 to D40. AP1903 (blue line), CAR T cells (red line). ***p < 0.01.

Because the IL-1RAP^low^ CAR+ HL-60 AML cell line was insensitive to CAR T cell cytotoxicity, we studied the in vivo activity of IL-1RAP CAR T cells against an IL-1RAP Luc+ or Luc+/CAR+ IL-1RAP^low^ HL-60 AML cell line in a xenografted NSG-S mouse model that received an injection of either HL-60-Luc+ or HL-60 CAR+ Luc+ cells. Compared with the untreated mice or those treated with T cells, mice treated with IL-1RAP CAR T cells displayed a decrease in leukemic burden of untransduced HL-60 cells, leading to complete elimination after day 7. In contrast, in mice that received IL-1RAP CAR+ HL-60 cells, no anti-leukemic effect of CAR T cells was detected in either mock-T cell- or IL-1RAP-CAR T cell-treated mice (Fig. 4).

Both in vitro and in vivo results suggest that IL-1RAP CAR expression on the cell surface of transduced leukemic AML cells enables their escape from cytotoxicity due to masking of the IL-1RAP antigen only when antigen cell surface expression is low, so that all the antigenic sites are bound by the CAR. On IL-1RAP^inter^ and IL-1RAP^high^ transduced leukemic AML cells, there are enough unmasked antigen sites expressed at the cell surface to render the cells sensitive to killing by the CAR T cells.

### A safety switch (suicide gene system iCASP9/Rimiducid) allows resistant CAR-transduced AML IL-1RAP^low^ cells to be eliminated, in vitro and in vivo

The IL-1RAP CAR vector includes an iCASP9 transgene. We took advantage of the insensitivity of IL-1RAP^low^-transduced IL-1RAP CAR AML cells to killing by the CAR T cells to test the usefulness of the safety switch in this model of epitope masking.

For an in vivo test, NSG-S mice were engrafted with only IL-1RAP^low^-transduced IL-1RAP CAR HL-60 AML leukemic cells i.v. on day 0. AP1903 or PBS was administered i.p. to the treatment and control groups, respectively, on day 3 (Fig. 4). As expected, the HL-60 AML tumor is controlled by IL-1RAP CAR T cells, whereas AML IL-1RAP CAR+ HL-60 tumor is not. In addition, if AP1903 treatment alone has no effect on untransduced HL-60 cell line and progressed, the AML HL-60 CAR+ tumor decreased in size after AP1903 injection until it disappeared. Leukemic transduced cells were not eliminated after PBS treatment (data not shown).

**Fig. 5:**
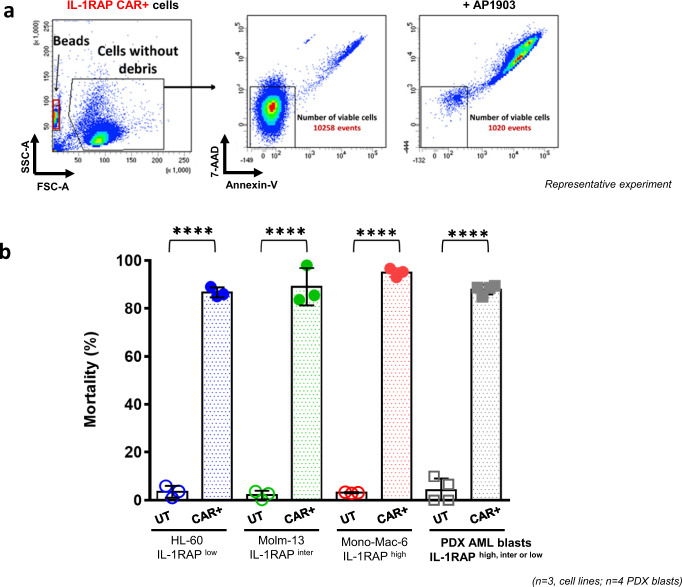
iCASP9 suicide system functional assay with the IL-1RAP^low,inter or high^ CAR+ leukemic AML cell lines or patient-derived xenograft (PDX) AML blasts. Untransduced (UT, open symbols, clear bars) or CAR-transduced (CAR+, closed symbols, grey bars) leukemic AML cells were cultured in medium alone or medium containing 20 nM Rimiducid. After 24 h of Rimiducid exposure, cells were collected and transferred to Trucount tubes and stained with anti-CD19, anti-Annexin-V, and 7-aminoactinomycin D (7-AAD). **a** Gating strategy for cytometry for discrimination of viable from dead cells. The quantification was performed after acquiring 5000 fluorescent beads. The mortality rate was normalized to control cells (untreated cells) and calculated as follows: % Dead cells = [1 − (absolute number of viable cells in AP1903-treated cells/absolute number of viable cells in untreated cells)] × 100. **b** Mortality results are shown as mean ± SD from triplicate (cell lines) or quadruplicate (PDX AML blasts) samples for each condition. ****p < 0.0001.

In vitro, a 24-h AP1903 (Rimiducid) treatment of IL-1RAP ^low^ CAR+ HL-60, IL-1RAP^inter^ CAR+ Molm-13, IL-1RAP^high^ CAR+ Monomac-6 AML cells, and CAR+ IL-1RAP^low,inter or high^ PDX AML blasts eliminated >85% of the cells (Fig. 5a, b), independently of IL-1RAP expression levels.

These results indicate that AP1903 or Rimiducid treatment induced effective and significant elimination of the transferred transduced tumor cells by activation of the iCASP9 suicide gene safety switch.

## Discussion

CAR T cell immunotherapies have proven to be remarkably efficient in treating B hematological malignancies in R/R patients by targeting the CD19 antigen. This success has been emphasized because it offers an alternative to patients at a therapeutic impasse until probable long-term remission. However, it should be noted that a percentage of patients are refractory to CAR T cells early in treatment or relapse late [26]. These failures can be assigned to intrinsic (expression of non-human transgenes, T cell population subtypes, kind of activating signal) or extrinsic (interaction with microenvironment) factors affecting CAR T cell efficiency. Tumor cells can also develop mechanisms to escape from the cytotoxicity of engineered T cells, remaining antigen positive (when they express an immune checkpoint or disrupting death receptor signaling [27]) or becoming antigen negative through CD19 splicing or mutations, lineage switching, or trogocytosis. Ruella et al. [14] described and explained escape due to a single B-ALL tumor cell transduction event leading to membrane expression of CAR and consequent auto-masking of the cell surface antigen by the expressed CAR itself, which blocked tumor cell recognition by the CAR T cells. There are several molecular and pharmacological on/off safety switches for CAR T cell immunotherapies that reversibly or irreversibly affect CAR T cells [28], but they cannot overcome adverse events involving tumor cells.

Today, major CAR T cell therapies are autologous, and effector cells are usually harvested from the patient at the time of relapse and thus include a large number of tumor cells [29]. Collecting a large number of tumor cells in cytapheresis increases the risk of unintentionally genetically modifying them, after which they may persist throughout the manufacturing process and be infused into the patient. To monitor samples for the presence of tumor cells, the use of highly powerful cytometry or molecular assays used to detect minimal residual disease will be essential in order to identify residual tumor cells at the end of the production process.

Ex vivo manufacturing conditions using medium suitable for T cell activation and expansion but unfavorable for tumor cell growth can limit the occurrence of this unexpected adverse transduction. However, lentivirus vectors can efficiently transduce non-proliferating or slowly proliferating cells, such as CD34+ stem cells [30]. A better selection of starting biological product using either anti-CD3/CD28 bead activation for CD3+ cell selection or specific immunomagnetic CD4+ and CD8+ sorting [31] will avoid bringing tumor cells into the production process.

In the case of epitope masking after tumor cell transduction, there are several options available to overcome antigen loss and circumvent immune escape from CAR T cell therapies. First, multiple antigen targeting will reduce the probability of both epitope masking, using two different CARs that are co-administered or cotransduced, or encoded in a bicistronic construct [32], or by using a two-antigen-binding domain carried by the same construct with or without T cell activation domain dissociation (tandem CAR T cells) [16]. Another option proposed by Ruella et al. consists of a cellular antidote anti-CAR involving a second CAR targeting the first anti-CD19 scFv expressed by both the T cell effectors and the transduced tumor cells [19]. However, this attractive approach can be applied only to the approved CD19 CAR, which lacks safety switches, and would be difficult, time-consuming, and expensive to implement.

**Fig. 6:**
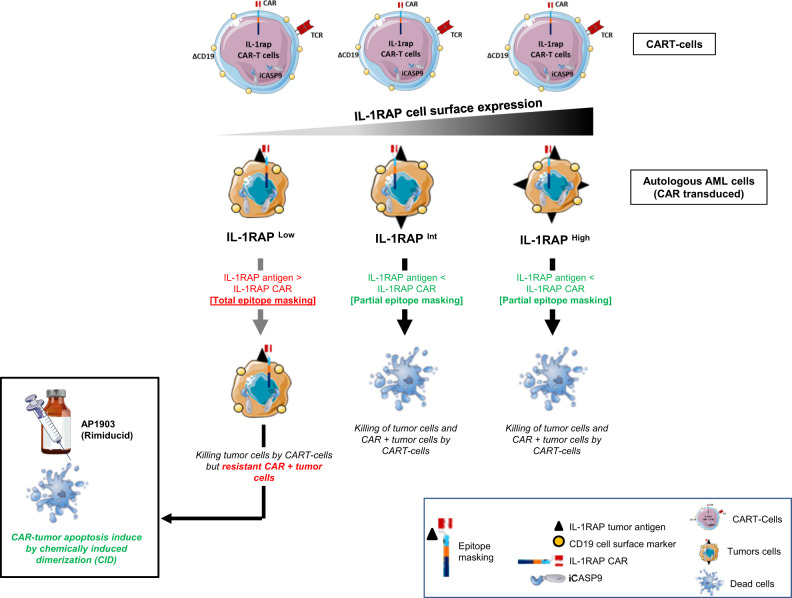
Summary model of the iCASP9 safety switch in autologous AML tumor escape induced by epitope masking after unexpected transduction of leukemic cells. Transduced IL-RAP CAR+/IL-1RAP antigen-expressing AML tumor cells are eliminated by IL-1RAP CAR T cells when the number of IL-1RAP antigen sites remains greater than the number of IL-1RAP CAR+ surface molecules (partial epitope masking). In case of total epitope masking after CAR transduction of AML tumor cells expressing low levels of IL-1RAP antigen, AP1903 (Rimiducid) exposure eliminates the cells that are resistant to CAR T cell immunotherapy.

In this work, we showed that epitope masking may occur in AML and demonstrated the usefulness of the suicide iCASP9/AP1903 system in this context, applying it to our original immunotherapy approach targeting IL-1RAP expressed by CML and AML leukemic cells [22]. While it is known that AML leukemic cells express different levels of IL-1RAP [25], by modeling using cell lines we were able to demonstrate that epitope masking occurs on cells expressing relatively low levels of the target, which can effectively be bound up by the CAR expressed on the membrane (total epitope masking). In other cases, the expression of antigen molecules at the cell surface is greater than CAR expression (partial epitope masking), and enough free antigen remains to trigger IL-1RAP CAR T cytotoxicity against the leukemic cells (Fig. 6).

For total epitope masking, we demonstrate that an on-board lentiviral construct safety switch, such as a suicide gene like iCASP9/AP1903 [20, 23], tEGFR/Cetuximab [33], or CD20/Rituximab [34], can eliminate CAR-transduced tumor cells that escape CAR T cell cytotoxicity. The advantage of a suicide gene safety switch is double, because it can also selectively ablate the CAR T cells when adverse events such as cytokine release syndrome, persistent neurotoxicity, on-target/off-tumor responses, or anaphylaxis occur.

## Conclusions

In conclusion, IL-1RAP CAR T cells produced from CD4+ and CD8+ selected cells and carrying an iCASP9 safety switch will strengthen the safety of our CAR T cell immunotherapy and will be applied in a future phase I clinical trial in AML.
